# Cutaneous Vasculitis: An Unusual Presentation of a Biclonal Nodal Plasma Cell Dyscrasia

**DOI:** 10.1155/2017/8152610

**Published:** 2017-09-13

**Authors:** D. Swan, M. Murphy, E. Elhassadi

**Affiliations:** ^1^Haematology Department, University Hospital Waterford, Regional Cancer Center South East, University College Cork, Cork, Ireland; ^2^Pathology Department, University Hospital Waterford, Regional Cancer Center South East, University College Cork, Cork, Ireland

## Abstract

We describe an unusual case of a biclonal nodal plasma cell dyscrasia, presenting with a vasculitic rash, end-organ damage, and cytopenias. Serum protein electrophoresis demonstrated a biclonal kappa-restricted paraprotein, with a negative skeletal survey and no bone marrow disease. Fluorodeoxyglucose-PET-CT (FDG-PET-CT) revealed nodal involvement, which was not appreciable clinically, and facilitated biopsy, confirming the diagnosis of a nodal plasmacytoma. Complete biochemical response and resolution of the vasculitic rash were achieved with bortezomib-based therapy.

## 1. Introduction

The incidence of multiple myeloma in the Republic of Ireland is around 5/100,000, with around 240 new cases presenting annually. Here, we discuss a challenging case of a biclonal nodal multiple myeloma, presenting with a vasculitic rash. Nodal and biclonal myelomas are individually extremely unusual, and the combination of both is vanishingly so. To date, and to the best of our knowledge, there have been no reported cases presenting with a vasculitic rash, a unique feature in our patient. This rare and unusual presentation of a condition seen commonly within the field of haematology required an individualised approach to care and necessitated modification of the routine investigational pathway recommended by the British Society of Haematology (BSH) [1].

## 2. Case Presentation

A 69-year-old lady was referred from the dermatology service in May 2016 for assessment of thrombocytopenia and rash. The rash was predominantly localised to the right leg and lower back and developed 3 weeks after ipsilateral total knee replacement surgery. She had normochromic, normocytic anaemia (Hb: 9.4 g/l), thrombocytopenia (105 × 10^9^/l) with prominent rouleaux on peripheral blood film, and moderate renal impairment without hypercalcaemia, with a normal erythrocyte sedimentation rate (ESR). Serum protein electrophoresis demonstrated a biclonal phenotype with a total IgG of 29.2 g/l, IgM of 0.44 g/l, and IgA of 33.8 g/l, with an IgA kappa band of 30.3 g/l and a small unquantifiable IgG kappa band. The absolute kappa free light chains were 259.08 mg/l, lambda free light chains were 48.72 mg/l, and the involved : uninvolved free light chain ratio was 5.32 (normal reference ranges: IgG 7–16 g/l, IgM 0.4–2.3 g/l, IgA 0.7–4.3 g/l, kappa free light chains 3.3–19.4 mg/l, lambda chain 5.71–26.3 mg/l, and ratio 0.26–1.65). Bence-Jones protein analysis was negative and beta-2-microglobulin was significantly elevated, at 10.48 mg/l. The X-ray skeletal survey was negative for lytic lesions.

**Figure 1:**
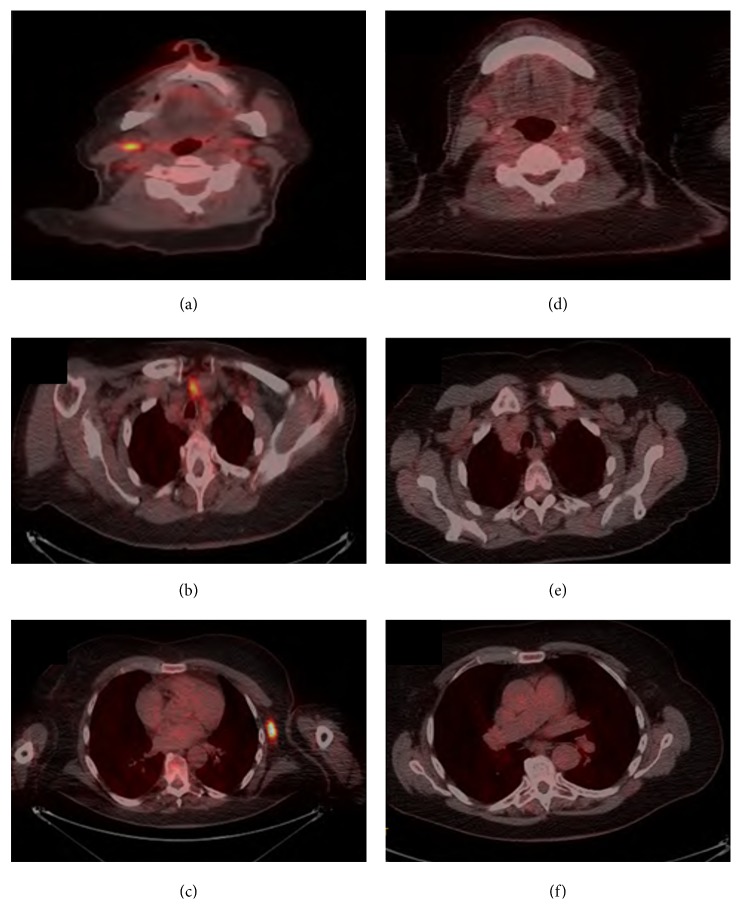
(a, b, c) Diagnostic axial FDG-PET images illustrate FDG-avid axillary, mediastinal, and cervical nodes, respectively. (c, d, f) Posttreatment axial PDG-PET images illustrate no FDG-avid evidence of residual disease at cervical, mediastinal, and axillary nodes, respectively.

**Figure 2:**
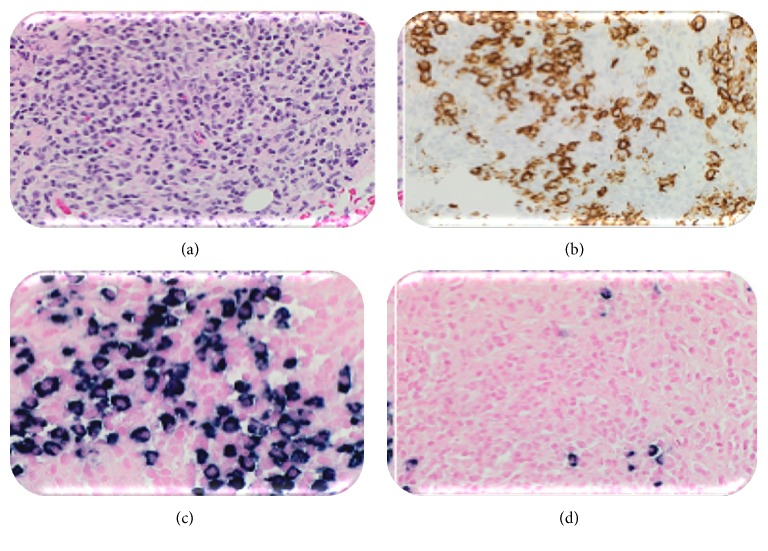
Left axillary lymph node biopsy. (a) Haematoxylin and eosin stain at ×40. (b) CD138 stain at ×40, strongly positive. (c) Kappa stain at ×40, strongly positive. (d) Lambda stain at ×40, negative demonstrating kappa light chain restriction.

Initial bone marrow examination demonstrated fewer than 10% plasma cells with no light chain restriction to suggest clonality. A repeat sample obtained from the contralateral iliac crest also contained fewer than 10% plasma cells, but these were kappa-restricted. Skin biopsy demonstrated changes consistent with the diagnosis of vasculitis. A fluorodeoxyglucose-PET-CT (FDG-PET-CT) was performed to localise the paraprotein-producing tumour, demonstrating bilateral FDG-avid cervical and axillary nodes, with a single mediastinal and portacaval node (Figures 1(a), 1(b), and 1(c)). The largest axillary node (7 mm) was biopsied revealing replacement of the nodal architecture by a CD138 positive, kappa-restricted plasma cell infiltrate (Figure 2).

The patient was started on CyBorD chemotherapy (cyclophosphamide, bortezomib, and dexamethasone) with symptomatic improvement and resolution of the vasculitic rash. The paraprotein present in the beta region reduced to 7.2 g/l and the IgA kappa band became undetectable. Free light chain ratio normalised to 1.40 with an absolute kappa of 75.53 mg/l and lambda of 54.11 mg/l. Her anaemia significantly lessened, and thrombocytopenia resolved between chemotherapy cycles. A very good partial response (VGPR) was achieved according to the International Myeloma Working Group (IMWG) criteria. Moreover, repeat FDG-PET-CT following 4 cycles of treatment showed a complete response (Figures 1(d), 1(e), and 1(f)).

## 3. Discussion

This case is noteworthy in three regards: the presentation with a vasculitic rash, the biclonal phenotype of gammopathy, and the extramedullary lymph node presentation without significant bone marrow involvement. In addition to this, the emerging and developing role of PET-CT in the management of multiple myeloma has clear relevance to this case.

Paraneoplastic vasculitis is a well-documented but poorly understood phenomenon, which is known to be more common amongst patients with haematological malignancies. A recent study of 421 patients with cutaneous vasculitis identified malignancy in 3.8%, of which over half were haematological in origin [2]. The authors suggested factors that should prompt investigation for underlying neoplasm including constitutional symptoms, abnormal circulating cells on peripheral blood smear, cytopenias, an elevated ESR, lymphadenopathy or organomegaly on clinical examination, and masses on imaging. Of the 9 patients in this study who were found to have a haematological malignancy, 100% were anaemic at the time of diagnosis, compared with 29% of patients with nonhaematological cancers and only 19% of those in whom a cancer diagnosis was not made. In this study, thrombocytopenia was reported in 12.5% of cases diagnosed with malignancy and <1% without. Our case presented with fatigue and investigations revealed bicytopenias and normal ESR; however, it should be noted that systemic corticosteroid therapy had been instigated by the dermatology team prior to our review, which could have mitigated this.

Biclonal multiple myeloma is a rare form of myeloma, with frequencies reported in the literature from 1 to 5%. A historical case series of 57 patients noted that 53% had an IgG and IgA band, and of 115 light chains reviewed, 70% were kappa [3]. Our case conformed to these observations. Recent work on the development of symptomatic myeloma from biclonal gammopathy of uncertain significance has not shown a greater risk of disease progression, or a poorer response to treatment in those that made progress [4], and there is no evidence to suggest that a different approach to therapy is warranted at the current time in those without evidence of organ damage or presence of high risk biomarkers. Development of a second clone following myeloma treatment does not also appear to impact outcome. Data on outcomes in those with symptomatic biclonal myeloma, such as our patient, is even more spare. Two cases of IgD/IgM myeloma have been reported, both with aggressive, chemotherapy-resistant behaviour [5], but to our knowledge, there is no robust evidence for those with symptomatic IgG/IgA disease.

Primary extramedullary plasmacytoma is also rare, accounting for only around 4% of plasma cell dyscrasias. There is some data suggesting that the risk of progression to multiple myeloma from a solitary primary lymph node plasmacytoma is less than for other extramedullary plasmacytomata and that recurrence posttreatment is rare [6], but evidence is limited to small case series and individual case reports. In contrast to this, patients with symptomatic extramedullary myeloma at diagnosis are more prone to high risk cytogenetic profiles, aggressive disease, and worsened outcomes [7, 8]. Optimal treatment is contentious, but there is some evidence that bortezomib-based therapies are efficacious in this setting and have been recommended by expert opinion [9, 10]. Our patient had constitutional symptoms, cytopenias, and renal impairment; hence, we felt that a bortezomib-based regime was an appropriate choice.

The role of FDG-PET-CT in the diagnosis and follow-up of myeloma is developing. At present, routine use of PET is not recommended in the most recent BSH guidelines due to insufficient evidence [11]. However, subsequent to our initial management of this case, in April 2017, the International Myeloma Working Group released a consensus statement on the role of FDG-PET in the diagnosis and management of multiple myeloma and other plasma cell disorders [12]. They recommend mandatory PET-CT to confirm suspected solitary plasmacytoma, if whole body MRI is unavailable, and to distinguish active from smouldering myeloma if skeletal survey is negative and whole body MRI is not possible. Here, we utilised PET-CT in order to localise the paraprotein-producing tumour and to facilitate confirmatory histological biopsy.

A meta-analysis of 14 studies found PET-CT to have superior specificity and sensitivity (96% and 77%, resp.) for the detection of sites of clonal plasma cells, particularly in extramedullary locations [13]. Mulligan and Badros reported that PET-CT is particularly useful for localisation of extramedullary disease, revealing additional lesions in around 30% diagnosed with a solitary plasmacytoma on MRI [14]. This feature benefited our case both diagnostically, as she lacked palpable nodes, masses, or size-significant lymphadenopathy by nonfunctional imaging modality criteria, and equally in terms of assessing response to treatment, for the same reasons. A lack of consensus currently exists regarding cut-off maximum standardised uptake values to distinguish positive from negative scans. In 2016, an Italian group proposed PET-CT criteria for use in myeloma at diagnosis and during treatment [15], which would include assessment of bone marrow uptake, osteolytic lesions, fractures, and extramedullary and paramedullary diseases. This may form the backbone of future reporting criteria.

The unusual nature of this case has meant that it has been both diagnostically and therapeutically challenging, with little robust evidence available to guide decision-making, as there are no randomised controlled studies focusing on patients with either biclonal myeloma or nodal disease. It highlights that the presence of a vasculitic rash should prompt consideration of underlying malignancy in certain patients, particularly those with anaemia or other cytopenias. Additionally, it demonstrates the emerging role of PET-CT in the management of plasma cell dyscrasias, with particular value in the setting of extramedullary disease.
